# Transient gingival inflammation is associated with epithelial dysfunction and systemic immune activation beyond clinical improvement

**DOI:** 10.1002/jper.70028

**Published:** 2026-05-13

**Authors:** Omnia Elebyary, Rachel Liu, Jinny Tsang, Chunxiang Sun, Bryan Coburn, Michael Glogauer

**Affiliations:** ^1^ Faculty of Dentistry University of Toronto Toronto Ontario Canada; ^2^ Dental Oncology Princess Margaret Cancer Centre Toronto Ontario Canada; ^3^ Department of Medicine University of Toronto Toronto Ontario Canada; ^4^ Department of Immunology University of Toronto Toronto Ontario Canada; ^5^ Division of Infectious Diseases University Health Network Toronto Ontario Canada

**Keywords:** epithelial barrier, gingivitis, immunoglobulins, polymorphonuclear leukocytes

## Abstract

**Background:**

The oral cavity significantly influences systemic health. Gingivitis, an early reversible stage of periodontal disease, may trigger systemic immune changes by facilitating bacterial translocation. This study examined the immune responses elicited by experimental gingivitis and their systemic immune impact, specifically in relation to the oral commensal Viridans group streptococci (VGS).

**Methods:**

Thirteen healthy participants (aged 18–55 years) underwent an experimental gingivitis model. Participants ceased oral hygiene practices for 3 weeks to induce gingival inflammation, followed by a 3‐week resolution period with resumed oral hygiene. Blood and oral samples were collected at 4 timepoints to measure epithelial barrier function markers, polymorphonuclear leukocytes (PMNs) counts, and activation marker expression, as well as total and anti‐VGS immunoglobulin IgG and IgA levels in the serum, which were measured using flow cytometry and enzyme‐linked immunosorbent assay.

**Results:**

Gingivitis induction led to significant increases in clinical parameters, which were nearly, but not completely, restored after resuming oral hygiene. Serum sCD14 and LBP levels (markers of epithelial integrity) increased in the gingivitis phase, and sCD14 remained high after the recovery phase on day 42 (*p* < 0.05). Similarly, PMN counts and surface marker expression in the blood were significantly upregulated during inflammation. PMNs remained primed, displaying higher responses to stimulation on day 42. Total serum IgG and IgA levels increased (*p* < 0.05), with elevated Anti‐VGS IgA levels that were borderline significant (*p* = 0.06).

**Conclusion:**

Experimental gingivitis triggers immune responses that remain elevated during the early phase of clinical improvement. Elevated levels of sCD14 and heightened PMN activity indicate ongoing subclinical inflammation and epithelial barrier dysfunction.

**Plain language summary:**

The mouth is connected to the rest of the body—and has broader implications for overall health—than many people realize. This study explored how gingivitis, a common and reversible form of gum disease, can trigger changes that extend beyond the mouth. Thirteen healthy adults temporarily stopped brushing and flossing for 3 weeks to develop gingivitis, then resumed their oral hygiene for another 3 weeks. During this period, we collected blood and saliva samples to monitor immune responses. We found that even after participants resumed oral hygiene and their gums appeared mostly healed, some immune markers remained elevated, suggesting that low‐level inflammation persisted. Immune cells in the blood became more active, and levels of antibodies, especially those targeting common mouth bacteria, increased. These findings indicate that even mild gum disease can have lasting effects on the body's immune system and potentially influence overall health.

## 1 INTRODUCTION

The oral cavity plays a crucial role in influencing overall systemic health.^1^, ^2^ Periodontitis, characterized by irreversible damage to the supporting structures of teeth, disrupts the oral mucosal barrier, facilitating bacterial translocation into the bloodstream and driving systemic immune alterations.^3^, ^4^ These changes are associated with heightened neutrophil activity and increased inflammatory markers, thereby linking periodontitis to broader systemic diseases.^5^, ^6^, ^7^, ^8^ Although the systemic impacts of periodontitis are well‐documented, they have largely been assessed in cross‐sectional studies after periodontitis is established. The irreversible nature of periodontitis limits the ability to longitudinally investigate its impact on humans.

Gingivitis—an earlier, reversible stage of periodontal disease—causes transient gingival inflammation. Although periodontitis is irreversible and known to induce lasting mucosal and immune alterations,^5^, ^9^, ^10^, ^11^ the effects of gingivitis are less well understood. It remains unclear whether gingivitis results in similar measurable mucosal and immune changes, including cellular and antibody‐mediated responses, and if these changes fully resolve after clinical inflammation subsides. Gingivitis is uniquely suited for study through experimental models, where healthy individuals abstain from oral hygiene for 3 weeks, leading to plaque accumulation and inflammation, followed by a period of resumed oral care to allow the inflammation to resolve.^12^ This model allows for controlled longitudinal assessment of the progression and resolution of gingival inflammation. Previous studies using this model have demonstrated that at peak gingival inflammation, peripheral blood polymorphonuclear leukocytes (PMNs) exhibit increased activity.^5^, ^13^ Another study has shown that, as gingivitis develops, chemo­attractant levels in the gingival crevicular fluid rise and remain elevated even after the clinical signs of inflammation have resolved, implying that immune alterations may persist after the inflammation clinically resolved^14^.

In this study, we used this experimental gingivitis model to longitudinally assess changes in local (mucosal) and systemic innate and adaptive immune changes relevant to periodontitis. We hypothesize that gingivitis‐induced inflammation compromises mucosal barrier function and triggers systemic immune responses. These changes can potentially persist beyond clinical resolution, linking gingivitis, similar to periodontitis, to systemic diseases. To explore this, we specifically investigated humoral responses against Viridans group streptococci (VGS), a prototypical oral commensal that plays a dual role in oral health and systemic disease. Given its frequent implication in conditions such as infective endocarditis and bloodstream infections, VGS represents a compelling marker for tracking mucosal barrier leakage and systemic exposure to oral bacteria. We aimed to uncover novel insights into how even mild, reversible gingival inflammation might influence systemic immune responses, thereby challenging the traditional perception of gingivitis as a localized condition with limited systemic impact.

## 2 MATERIALS AND METHODS

### 2.1 Clinical study design

Thirteen healthy participants aged between 18 and 55 years were recruited from the University of Toronto, Faculty of Dentistry, including 7 males (53.8%) and 6 females (46.2%). The inclusion criteria were:(1) age 18 or older,(2) fluency in English, and(3) absence of active oral lesions defined as visible ulcerations, erosions, or mucosal pathology at the time of examination. Exclusion criteria included:(1)active or history of periodontitis,(2) presence of systemic conditions that could affect immune function or pose a risk during probing (e.g., diabetes, autoimmune diseases, neutropenia, or hypertension), (3) use of medications known to influence gingival bleeding or wound‐healing (such as systemic anticoagulants) which can inflate bleeding‐on‐probing and other periodontal charting measures, thereby confounding clinical assessments, and (4)smoking. Race and ethnicity were not recorded for this cohort. The study was explained to each participant, and informed consent was obtained before inclusion.

Oral and periodontal health was confirmed based on a complete dental examination according to current guidelines^15^ and as previously described.^16^ Oral examination included evaluation of soft and hard palate, buccal mucosa, mucogingival folds, tongue, sublingual and submandibular areas, salivary glands, and tonsillar and pharyngeal areas. Periodontal examination included recording periodontal probing depth at 6 sites per tooth, Löe–Silness gingival index (GI),^17^ visible plaque index (VPI),^18^ and bleeding on probing (BOP) at 6 sites per tooth. This study was approved by the University of Toronto Research Ethics Board (REB #33085) and conducted in accordance with the Declaration of Helsinki.

This human experimental gingivitis cohort was designed as described previously.^5^, ^13^ This study was 9 weeks long with 3 phases: pre‐induction, induction, and resolution. In the pre‐induction phase, participants were given oral care products and instructions (brush twice daily with a soft manual toothbrush) for 3 weeks to reduce confounding due to oral hygiene‐related variables. Day 0 (Baseline) marks the beginning of the induction phase, where participants were instructed to cease all oral hygiene practices for 3 weeks to reach peak gingival inflammation on day 21 (Peak gingivitis). This was followed by a 3‐week resolution period where brushing and oral hygiene practices were reinstituted to achieve recovery by day 42 (Three weeks post). Blood and oral rinse were collected on Days 0, 21, 28, and 42 (Figure S1 in the online *Journal of Periodontology*).

Additionally, we recruited an independent cohort of patients from the Faculty of Dentistry clinics at the University of Toronto and stratified them into the following periodontal categories: Localized stage III periodontitis (*n* = 8), Generalized stage III periodontitis (*n* = 8), and Generalized stage IV periodontitis (*n* = 8). Peripheral blood samples were obtained from all participants for downstream immunological analyses.

### 2.2 Quantification of serum markers of epithelial disruption

Serum concentrations of epithelial dysfunction markers soluble CD14 (sCD14) and lipopolysaccharide binding protein (LBP) were assessed by a custom Luminex assay (1:400 dilution) according to the manufacturer's instructions. Luminex data were collected using xPONENT version 4.2 software on a Luminex MagPix machine (Luminex, Toronto, Canada). Outputs greater than the upper limit of the standard were adjusted to the top standard value. Outputs less than the lowest standard dilution concentration were adjusted to one‐third the value of the lowest standard.

### 2.3 Oral and blood polymorphonuclear neutrophil count and CD marker expression

Oral polymorphonuclear neutrophil (oPMN) counts were determined as previously described.^16^ Briefly, pooled saline oral rinses were fixed with formaldehyde (1.6% w/v final concentration) for 15 min on ice and filtered. Cell counts were acquired on a Coulter counter with a size range of 8–12 µm.

For blood PMNs, whole blood was fixed with fresh, methanol‐free formaldehyde (1.6% final concentration w/v) for 15 min on ice prior to processing. Red blood cells were lysed by repeated treatment with a lysing solution, BD Pharm Lyse buffer (BD Biosciences). Flow cytometer channel voltages were calibrated manually using rainbow beads, and compensation was performed with single‐stained compensation beads. At least 2 × 10^5^ gated events were acquired per sample. Data were analyzed using FlowJo software (v. 10; TreeStar). Human multicolor flow cytometry panels and gating were conducted as previously described.^19^ To determine the overall degree of activation of the PMNs, we looked at 6 CD marker signatures on PMNs that have previously been linked to PMN activation, recruitment, and pathogen killing. The markers were classified into 3 categories based on function: degranulation/activation markers (CD63, CD64, and CD66),^19^, ^20^ adhesion markers (CD11b and CD18),^21^ and the Fc receptor marker (CD16).^22^ Appropriate isotype control antibodies were used to generate fluorescence‐minus‐1 samples to establish negative staining for each antibody

To assess the functional responsiveness of blood PMNs, whole blood samples were stimulated ex vivo with N‐formylmethionine‐leucyl‐phenylalanine (fMLP). Briefly, 100 µL of whole blood was incubated with 10 µL of 100 µM fMLP at 37°C for 30 min. Following stimulation, samples were fixed with 1.6% w/v methanol‐free formaldehyde on ice for 15 min and processed as described above for flow cytometric analysis.

### 2.4 Total IgA and IgG quantification

Serum samples were diluted in phosphate‐buffered saline (PBS) with 1% Tween 20 w/v and 10% bovine serum albumin w/v and centrifuged at 10,000 x*g* for 10 min. Total immunoglobulin (Ig) A and IgG levels were quantified using human IgA and IgG (total) uncoated enzyme‐linked immunosorbent assay (ELISA) kits (Invitrogen) according to the manufacturer's instructions. The lower limit of quantification was determined using the lowest value from the standard curve across all plates analyzed and applied to all values below the lower limit of quantitation. Only samples above the upper limit of quantification were diluted and re‐analyzed. Internal laboratory controls were used as inter‐assay controls.

### 2.5 Bacterial preparations

*Streptococcus mutans* (NCTC 10449 [IFO 13955]), Streptococcus mitis (NCTC 12261), *Streptococcus sanguinis* (DSS‐10), *Streptococcus salivarius* (C699 [S30D]), and *Streptococcus anginosus* (NCTC 10713) were grown in Brain Heart Infusion broth aerobically (5% CO_2_) at 37°C for 17–20 h. Bacteria were harvested at a concentration of ∼1 × 10^9^ colony‐forming units (CFU) and stored in 20% glycerol in −70°C. When thawed, bacteria were washed with PBS, and all 5 species were combined at equal volumes to create a *Streptococcus* spp. consortium. This consortium was stained with CFSE (Thermo Fisher) for 30 min before incubating with clinical samples.

### 2.6 Microbe‐binding antibody assay

The anti‐VGS antibody protocol was adapted from Moor et al.^23^ Briefly, serum samples were diluted (1:160) with fluorescence‐activated cell sorting (FACS) buffer (2% bovine serum albumin [BSA], 5uM ethylenediaminetetraacetic acid [EDTA]‐PBS) and heat‐inactivated for 30 min at 56°C. Then, samples were centrifuged at 16,000 xg to remove pelleted debris. Then, 50 uL of serially diluted (1:4 dilutions in FACS buffer) serum was combined with an equal volume of bacteria (∼ 5 × 10^5^ CFU/mL) and incubated for 1 h at 4°C. Unbound antibodies were then washed off with FACS buffer (4000 xg, 10 min, 4°C). Antibody‐bound bacteria were then stained with secondary antibodies conjugated with fluorochromes: goat anti‐human IgG F(ab’)2 antibody (AF594, Jackson Immunology Inc.) and goat anti‐human IgA F(ab’)2 antibody (AF647, Southern Biotech) for 30 min before washing off unbound antibodies with FACS buffer (4000 x*g*, 10 min, 4°C). Antibody‐bound bacteria were fixed with 4% paraformaldehyde (PFA) for 20 min and washed with FACS buffer (4000 x*g*, 10 min, 4°C). All samples were analyzed using an LSR Fortessa (Becton Dickinson) with settings optimized for bacterial‐cell detection. Internal lab controls were included for each assay to ensure inter‐assay reproducibility. Flow cytometry analysis of samples was performed using FlowJo (Treestart Inc.), representative gating strategies are presented in (Figure S2 in the online *Journal of Periodontology*).

Geometric mean fluorescence intensity (MFI) values were extracted for each sample at each dilution. Bacteria incubated without clinical samples and stained with secondary antibodies were used as the negative control and subtracted from all samples. Sample dilution factors and their corresponding MFI values were log10 transformed and plotted with a linear regression using GraphPad prism to produce regression lines for each sample. Negative log10‐transformed values are assigned a 0 value. Responses were reported as the area under the curve (AUC) value for each regression line.

### 2.7 Statistical analysis

Longitudinal changes on days 21, 28, and 42 were evaluated against each participant's baseline measurements, allowing for the calculation of fold‐changes in PMN CD marker expression and addressing baseline variability across participants. A repeated‐measures mixed‐effects model was employed to accommodate for any missing data points and capture subject‐specific effects over time. Multiple comparisons were performed to detect significant differences among time points, with only significant comparisons plotted on the figures. Soluble immune factors and antibody concentrations were log10‐transformed. Error bars represent the standard deviation. All analyses and visualizations were conducted using GraphPad Prism (version 9.0.2).

## 3 RESULTS

### 3.1 Experimental gingivitis clinical data

**FIGURE 1:**
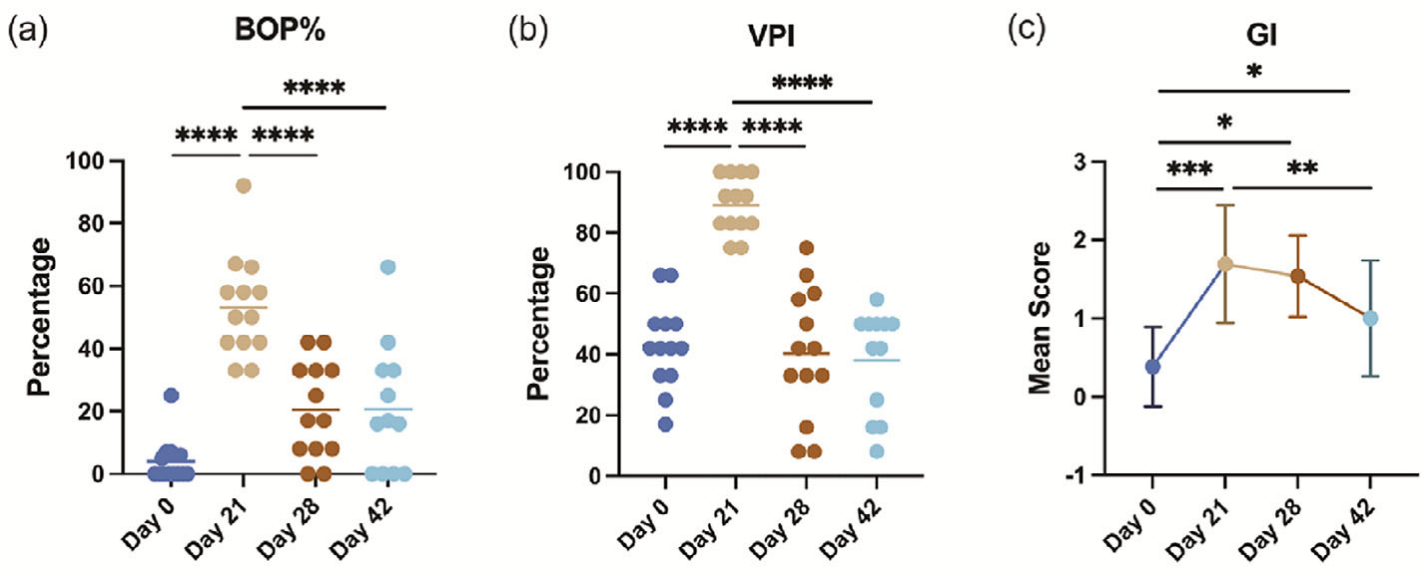
Clinical features BOP (a), VPI (b), and GI (c) were analyzed on day 0 (baseline), 21 (peak gingivitis), 28 (1 week post), and 42 (3 weeks post). Comparisons were made using a repeated‐measures mixed‐effects model with multiple comparisons between each of the timepoints. Only significant comparisons were plotted where: *p ≤ 0.05; **p ≤ 0.01; ***p ≤ 0.0005; ****p ≤ 0.0001. BOP, bleeding on probing; GI, gingival index; VPI, visible plaque index.

Participants in this study, with a mean age of 30.36 ± 2.40, displayed a peak in their gingival clinical parameters on day 21 during the induction phase. BOP demonstrated a 5‐fold increase from a mean of 10.53% on day 0 to 52.36% on day 21 (*p* < 0.0001) (Figure 1a). Similarly, the VPI and GI both exhibited an approximately 2‐fold increase in their mean values on day 21 compared to baseline (*p* < 0.0001 and *p* < 0.001, respectively) (Figure 1b,c). The levels of BOP and VPI were significantly reduced after resumption of oral hygiene and were not significantly different from baseline (Day 0); however, the GI of the participants remained slightly elevated (*p* < 0.05).

### 3.2 Markers of epithelial barrier dysfunction

**FIGURE 2:**
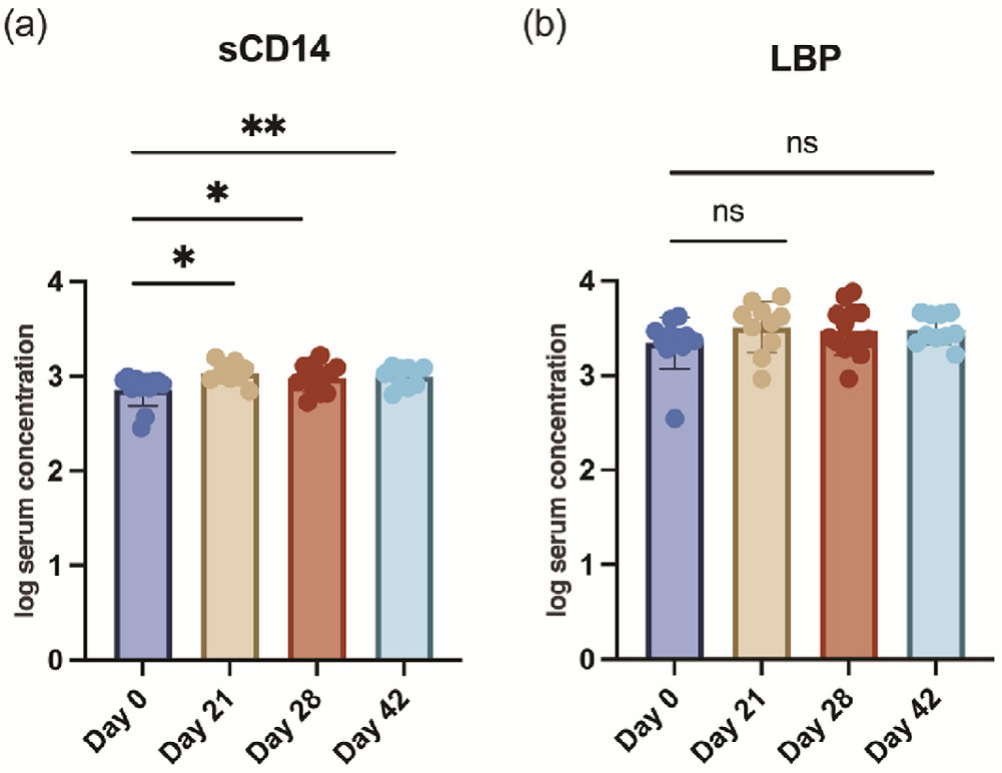
Markers of epithelial barrier dysfunction are elevated during and after experimental gingivitis. serum concentration distributions of (a) sCD14 and (b) LBP. Comparisons were made using a repeated‐measures mixed‐effects model with multiple comparisons between each of the timepoints. Only significant comparisons were plotted where: *p ≤ 0.05; **p ≤ 0.01.

To investigate persistent breakdown and leakage from the oral epithelium post‐gingivitis recovery, we measured serum concentrations of epithelial leakage markers sCD14 and LBP. Serum sCD14 levels significantly increased after gingivitis induction (*p* < 0.05) and peaked on day 42 (*p* < 0.01) and remained elevated even after a reduction in gingival inflammation was noted (Figure 2a). Although LBP levels followed a trend similar to that of sCD14, they did not show statistically significant changes, with a borderline significant increase on day 21 (*p* = 0.06) and remained relatively high on day 42 (*p* = 0.09) (Figure 2b). The persistently elevated sCD14 levels and the upward, though non‐significant, trend in LBP suggest that epithelial barrier integrity may remain compromised even after the clinical signs of gingival inflammation have largely resolved.

### 3.3 PMN counts in the oral cavity and blood

**FIGURE 3:**
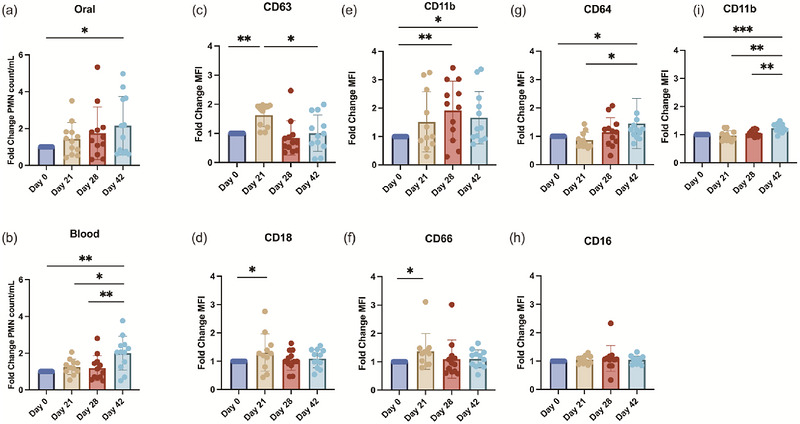
Fold change in PMN counts, gMFI for each of the CD markers in blood PMNs, and in CD11b in ex vivo stimulated blood PMNs, were analyzed. Comparisons were made using a repeated‐measures mixed‐effects model with multiple comparisons between each of the timepoints. Only significant comparisons (*p* < 0.05) are reported. Only significant comparisons were plotted where: *p ≤ 0.05; **p ≤ 0.01; ***p ≤ 0.0005. MFI, mean fluorescence intensity; PMNs, polymorphonuclear leukocytes.

To investigate changes in the innate immune response, we monitored changes in the predominant innate immune cells, PMNs. Blood and oral PMN counts did not increase significantly on day 21 (Figure 3a,b). However, by day 42, PMN counts were significantly upregulated at both media compared to baseline (oral: *p* < 0.05, blood: *p* < 0.01).

To determine the effect of gingival inflammation on PMN activity, samples were stained using a multicolor flow cytometry panel. The MFI of each CD marker was measured for the PMN population. On day 21, the PMN surface expression of CD11b, CD63, CD66, and CD18 were all significantly upregulated (Figure 3c–h). Nevertheless, upon marked improvement in clinical parameters, the surface expression levels of these markers returned to their baseline levels, with only CD11b and CD64 displaying significantly upregulated expression.

To assess PMN responsiveness over the course of the study, we stimulated isolated PMNs ex vivo with *fMLP* at each timepoint and measured surface CD11b expression. A heightened PMN response was observed on day 42, as evidenced by significantly increased CD11b expression compared to all other timepoints (*p* < 0.01) (Figure 3i). This upregulation indicates that circulating neutrophils remain in a primed, hyper‐responsive state even after clinical signs of gingivitis improve.

### 3.4 Total and anti‐VGS serum IgG and IgA

**FIGURE 4:**
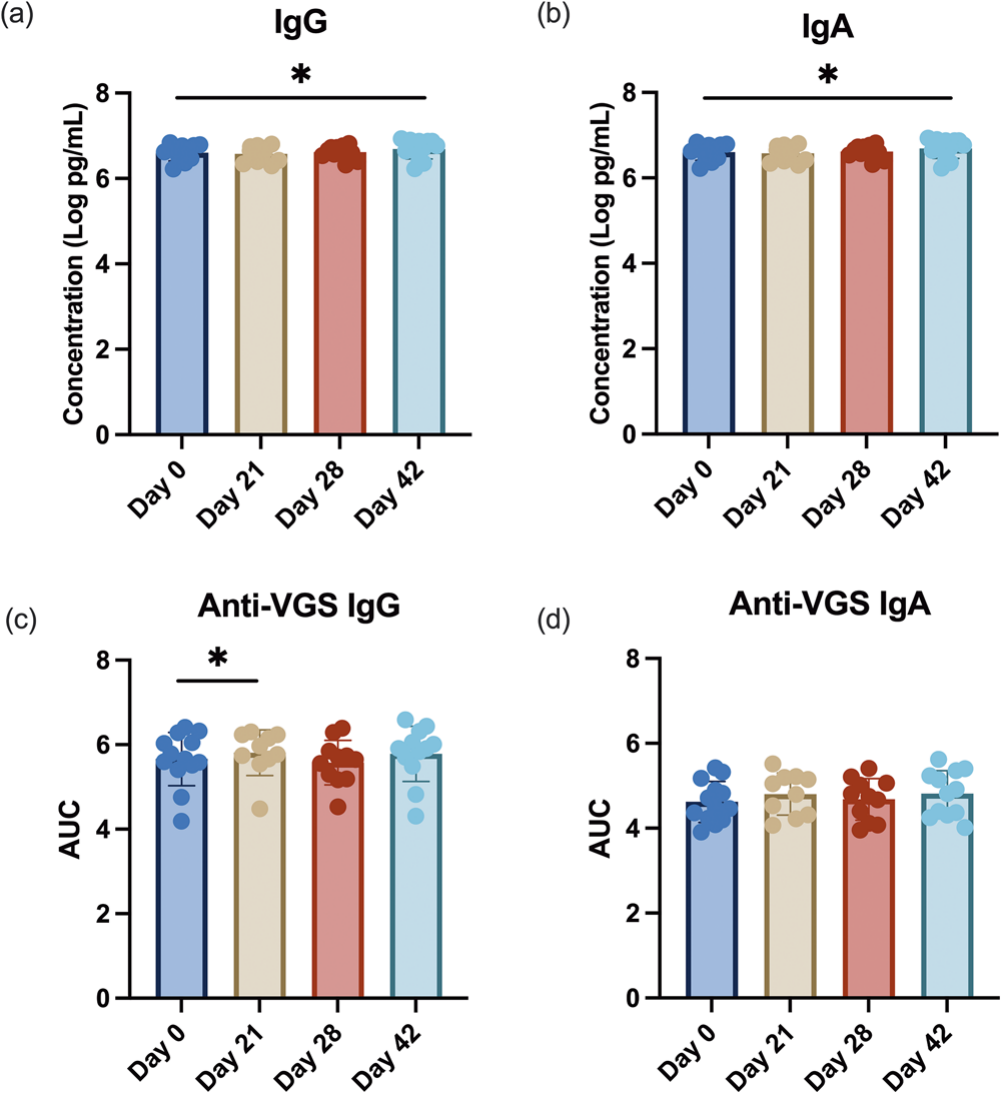
Total IgA (b) and IgG (a) in the serum and anti‐VGS IgA (d) and IgG (c) responses compared across the different timepoints. Repeated‐measures mixed‐effects model with multiple comparisons between each of the timepoints. Only significant comparisons were plotted where: *p ≤ 0.05. Ig, immunoglobulin; VGS, Viridans group streptococci.

Total serum IgG and IgA concentrations were measured. Although no significant changes were observed during the gingivitis induction phase, a significant increase in both IgA and IgG concentrations was noted on day 42 (*p* < 0.05**;** Figure 4a,b). This suggests a systemic immune response following gingivitis resolution.

Having shown that experimental gingivitis increases total serum IgA and IgG levels, we next evaluated whether this rise was accompanied by enhanced antibody responses specifically targeting VGS, key oral commensals. Indeed, on day 21‐elevated levels of anti‐VGS antibodies against VGS were observed. This change was significant for IgG (*p* < 0.05) and borderline significant for IgA (*p* < 0.1) (Figure 4c,d). After recovery, only Anti‐VGS IgA levels were elevated, but the difference was only borderline significant (*p* = 0.06).

### 3.5 Influence of periodontal inflammation severity on anti‐VGS responses

**FIGURE 5:**
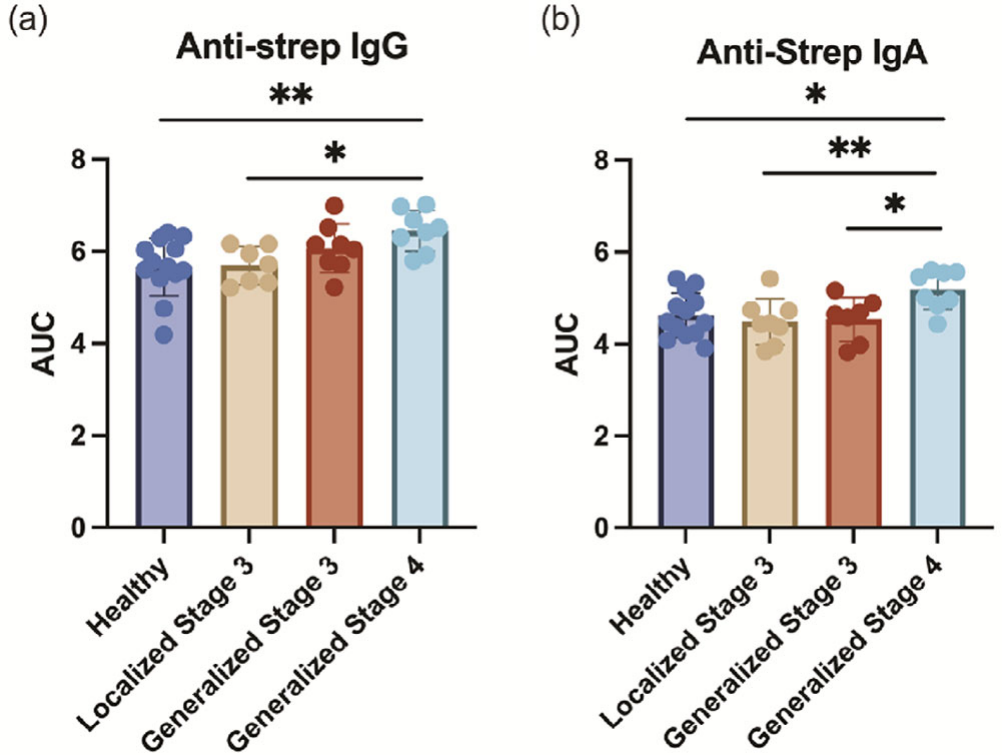
Anti‐VGS IgG (a) and IgA (b) targeting VGS compared across 4 different categories of participants (Healthy (*n* = 13), Localized stage 3 (*n* = 8), Generalized stage 3 (*n* = 8), and Generalized stage 4 (*n* = 8)). Differences between groups were analyzed using the Kruskal–Wallis test. Only significant comparisons were plotted where: *p ≤ 0.05; **p ≤ 0.01. Ig, immunoglobulin; VGS, Viridans group streptococci.

To extend our findings from reversible gingivitis to chronic disease, we examined anti‐VGS IgG and IgA levels in an independent cohort of participants presenting varying degrees of periodontitis (Periodontal Health, Localized stage III, Generalized stage III, Generalized stage IV). Our findings revealed that participants diagnosed with generalized stage IV periodontitis demonstrated more robust anti‐VGS IgG and IgA responses relative to those with generalized or localized stage III disease (Figure 5a, b). This observation supports our hypothesis that even commensal oral bacteria like VGS—typically overlooked in periodontitis studies—can elicit measurable systemic immune responses when the mucosal barrier is compromised.

## 4 DISCUSSION

This study provides novel longitudinal insights into the systemic consequences of gingivitis, revealing that even mild, transient oral inflammation may trigger measurable alterations in epithelial integrity, innate immune activation, and humoral responses. Although clinical signs showed marked improvement 3 weeks after oral hygiene resumption, full resolution was not achieved, and immune alterations persisted. This nuance is particularly important, as it contradicts the common assumption that experimental gingivitis is entirely reversible—an aspect often overlooked in studies that focus primarily on the induction phase. Our observations raise critical questions about the potential for prolonged or subclinical inflammation and its local and systemic implications for participants in experimental gingivitis models.

Despite the largely resolved clinical signs of gingivitis, including metrics of BOP, VPI, and GI metrics, a sustained elevation in serum sCD14 levels was observed following gingivitis, with levels peaking on day 42. sCD14, a co‐receptor for lipopolysaccharides (LPS), is a marker of epithelial dysfunction and systemic inflammation, and is commonly associated with conditions involving microbial translocation and barrier disruption.^24^, ^25^ Elevated sCD14 levels have been widely reported in severe periodontal diseases, reflecting ongoing epithelial leakage and systemic immune activation.^4^, ^26^, ^27^ Our study extends these findings by demonstrating that even gingivitis—typically considered a mild form of periodontal disease—can trigger systemic persistent evaluations in sCD14 levels, suggesting ongoing epithelial leakage.

Similarly, LBP, another marker of epithelial permeability and immune activation, exhibited borderline significant increases during the gingivitis induction and recovery phases. LBP binds to LPS, facilitating its recognition by immune cells and indicating increased epithelial permeability and systemic exposure to bacterial endotoxins.^28^, ^29^ Although LBP levels did not reach statistical significance, the trend closely mirrored that of sCD14. This discrepancy may reflect differences in their biological roles and clearance kinetics: LBP is an acute‐phase protein that binds circulating LPS early in inflammation and is rapidly cleared through mechanisms such as complex formation with high density lipoprotein (HDL) or sCD14.^30^, ^31^ As a result, LBP levels often return to baseline shortly after acute inflammation resolves, making it less sensitive to detecting low‐grade or sustained epithelial dysfunction. In contrast, sCD14 tends to remain elevated longer and may more reliably indicate ongoing microbial translocation.^32^ Taken together, the persistent elevation of sCD14 alongside the upward trend in LBP supports the presence of continued epithelial barrier disruption and systemic immune activation, even after visible clinical symptoms have improved.

When assessing the cellular responses to experimental gingivitis, we examined PMNs, the most abundant leukocytes in the circulation and the gingival tissues. Our previous investigation into their responses revealed significant changes in PMNs during gingivitis.^5^, ^13^ This study aimed to focus on the longer‐term implications and responses during the resolution phase. A recent study by Roberts et al. similarly examined this aspect using a split‐mouth 21‐day experimental gingivitis model.^14^ During the resolution phase of experimental gingivitis in their study, neutrophil extracellular trap (NET) and reactive oxygen species (ROS) production normalized following the reintroduction of oral hygiene practices. However, elevated levels of specific pro‐inflammatory mediators in gingival crevicular fluid remained, suggesting a subclinical inflammatory presence and the potential for a prolonged immune response that may continue to attract PMNs to the gingival tissues even after visible inflammation has resolved. Indeed, in this study, we found that oral and blood PMN counts had a 2‐fold increase at the recovery timepoint, consistent with previous reports where higher venous blood PMN counts were found in patients with gingivitis.^33^

Interestingly, we observed enhanced priming of blood PMNs at the time of recovery, as indicated by the upregulation of CD11b during ex vivo fMLP stimulation. This observation occurred 3 weeks post‐oral hygiene resumption, a period when most clinical inflammation had subsided. Similarly, oral and blood PMN counts remained significantly elevated at this time point. This sustained elevation might partly reflect persistent oral epithelial disruption or incomplete clinical resolution; however, the overall heightened neutrophil counts and responses despite reduced inflammatory burden strongly suggest the involvement of additional mechanisms. One potential explanation is the emerging concept of trained immunity, whereby innate immune cells—including neutrophils—can retain an “immunological memory” acquired through epigenetic and metabolic reprogramming of progenitor cells in the bone marrow.^34^, ^35^ Consequently, neutrophils produced under these conditions can mount more robust responses upon subsequent inflammatory challenges. Although this phenomenon has been previously documented primarily in the context of periodontitis,^36^, ^37^ our findings suggest that even milder forms of oral inflammation, such as gingivitis, can potentially induce similar innate immune memory effects. However, additional studies are necessary to substantiate this hypothesis.

The study also revealed significant changes in humoral immune responses, with increases in total serum IgG and IgA concentrations observed at the post‐recovery timepoint. This aligns with findings in periodontal disease, where immunoglobulins are elevated in response to systemic exposure to oral pathogens.^9^, ^38^, ^39^ Notably, our study is the first to investigate elevated levels of anti‐VGS antibodies, a group of dominant bacteria in the oral cavity. VGS, can act as both a commensal in the mouth and as pathogen with systemic translocation, serves as an intriguing target for assessing gingivitis‐associated humoral immunity. Elevated levels of anti‐VGS antibodies were detected on day 21, with significant increases in anti‐VGS IgG and borderline significance for anti‐VGS IgA. Although anti‐VGS IgA levels remained elevated post‐recovery, they did not reach statistical significance (*p* = 0.06), likely due to the small sample size. We observed more robust responses with more severe forms of periodontitis, highlighting systemic humoral responses to VGS exposure.

The findings of this study, though derived from a relatively small sample size over a short observation period, suggest that gingivitis may be more than just a mild, localized oral condition. The persistent immune activation observed points to the possibility that gingivitis could induce systemic inflammatory responses with potential implications for overall health, even after substantial but incomplete clinical resolution. An example of this is the altered responses toward VGS, which are frequently implicated in infective endocarditis and bloodstream infections in neutropenic patients.^40^, ^41^, ^42^ Such changes could modulate susceptibility to these infections. Nonetheless, further research with larger cohorts and extended follow‐up is necessary to confirm and expand upon these observations, clarify the underlying mechanisms, and fully determine the impact of gingivitis on systemic health, particularly regarding Streptococci‐related infections.

Despite the exciting findings, several limitations in this study must be acknowledged. The small sample size of thirteen participants may limit the generalizability of the findings. Additionally, while the study employed an experimental gingivitis model to precisely control the timeline of inflammation progression and resolution, this model may only partially capture the complexity and variability of gingivitis in a real‐world setting. In particular, adherence to oral‐hygiene instructions outside of a supervised clinical environment is known to vary considerably, which could introduce additional variability in clinical outcomes. Another limitation is the short follow‐up period; although significant immune alterations were observed post‐recovery, the study did not extend beyond 42 days, leaving long‐term effects unexamined. Moreover, reliance on sCD14 and LBP as indicators of oral barrier disruption does not exclude potential contributions from other sites, such as the gastrointestinal tract. Future research should address these limitations to provide a more comprehensive understanding of gingivitis and its long‐term implications.

Persistent epithelial leakage, ongoing PMN activation, and elevated humoral responses against key oral bacteria highlight the potential of gingivitis to influence systemic immunity and predispose individuals to related conditions. These findings challenge the traditional view of gingivitis as a localized and reversible condition, emphasizing the need for a broader perspective on its management and impact on overall health. Future research should focus on pinpointing the mechanisms that drive these immune alterations and developing targeted interventions to mitigate their systemic effects.

## AUTHOR CONTRIBUTIONS

All authors contributed to the study. Omnia Elebyary, Bryan Coburn, and Michael Glogauer conceptualized the study. Omnia Elebyary and Chunxiang Sun collected the samples. Omnia Elebyary, Rachel Liu, and Jinny Tsang performed the experiments. Omnia Elebyary, Bryan Coburn, and Michael Glogauer interpreted the data and drafted the manuscript.

## CONFLICT OF INTEREST STATEMENT

The authors declare no conflicts of interest.

## Supporting information



Supporting Information

## Data Availability

The data supporting this study's findings are available on request from the corresponding author.
