# Supplementary material for: Transient gingival inflammation is associated with epithelial dysfunction and systemic immune activation beyond clinical improvement
Source: J Periodontol. 2026 May 13;97(6):1147–56. doi: 10.1002/jper.70028 (PMC13350218; doi:10.1002/jper.70028)
Supplement: Supplementary file 1 — Supporting Information [file JPER-97-1147-s001.docx]

**Supplementary Figures:**


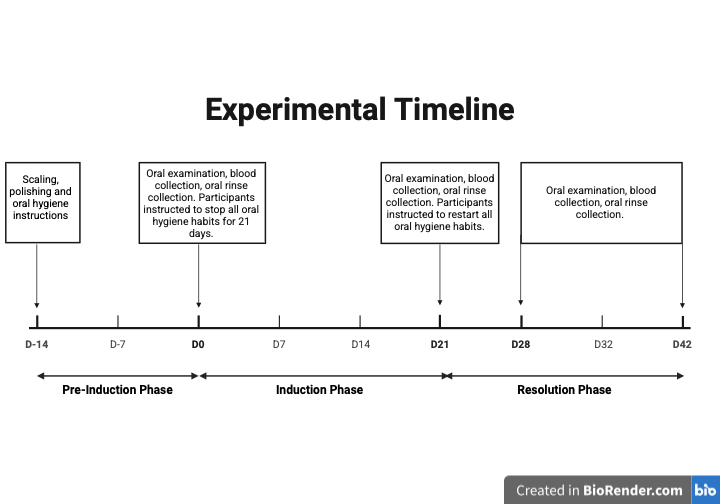


Supplementary Figure (1): Sample Collection and Processing


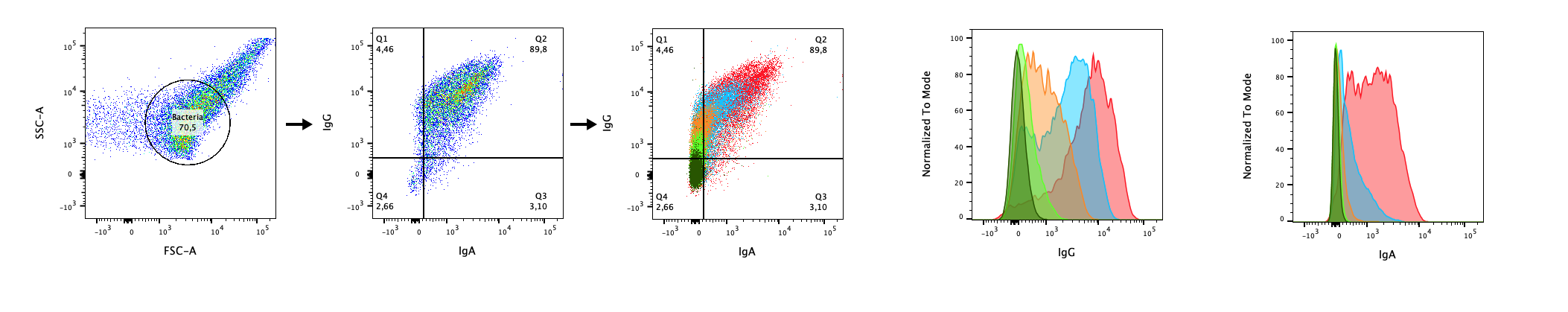


Supplementary Figure (2): Gating Strategy for Anti-VGS IgG and IgA on flow cytometry.
